# Potassium level changes in chronic kidney disease patients following balanced crystalloid administration in the emergency department

**DOI:** 10.1097/MD.0000000000035026

**Published:** 2023-09-29

**Authors:** Sungsig Kong, Hyuksool Kwon

**Affiliations:** a Department of Emergency Medicine, Seoul National University Bundang Hospital, Gumi-dong, Bundang-gu, Sungnam-si, Gyeonggi-do, Republic of Korea; b Department of Emergency Medicine, Seoul National University College of Medicine, Seoul, Republic of Korea.

**Keywords:** balanced crystalloid, chronic kidney disease patients, emergency department, potassium

## Abstract

One common reason why clinicians are often hesitate to administer balanced crystalloids in the emergency department is the potential occurrence of unexpected hyperkalemia in patients with chronic kidney disease (CKD). In order to investigate the changes in potassium levels resulting from the administration of balanced crystalloids, specially Plasma Solution A (a generic version of Plasma-Lyte), to emergency department patients with CKD, we conducted an evaluation. A retrospective cohort study was conducted at a single academic hospital. Our study included patients with CKD who received intravenous Plasma Solution A and underwent electrolyte follow-up testing within 24 hours of administration. In total, there were 745 patients included in this study, of whom 87 had CKD. Through a 1:1 propensity score matching procedure for factors other than the estimated glomerular filtration rate, we matched 87 patients with normal kidney function to 87 CKD patients. For patients with CKD, the mean standard deviation SD administered volume of Plasma Solution A was 28.7 (21.0) mL/kg, and the mean SD administration duration was 13.2 (4.5) hours. The mean SD potassium level decreased from 4.3 (0.6) mmol/L to 4.1 (0.6) mmol/L (P = .029). Our study findings suggest that there may be no significant harmful increase in potassium levels or worsening of renal function within 24 hours after the intravenous administration of approximately 2 L of Plasma Solution A to patients with CKD.

## 1. Introduction

Fluid administration serves as a crucial resuscitation tool in the emergency department (ED), aimed at restoring hemodynamic stability, replenishing fluid loss, and maintaining intravascular volume. Recently, a large-scale trial randomized intensive care unit (ICU) patients to receive either saline or balanced crystalloids.^[1]^ Patients administered with balanced crystalloids showed more favorable outcomes concerning mortality and major adverse kidney events, including dialysis and persistent renal dysfunction.^[2]^

However, a more recent study demonstrated no significant difference in outcomes for ICU patients, suggesting that the benefits of balanced crystalloid administration may not be significant to patients in the ICU requiring long-term intensive care.^[3]^ Nonetheless, a comprehensive ED study involving patients not admitted to the ICU showed significantly better outcomes in terms of death, new renal replacement therapy, and persistent renal dysfunction, favoring treatment with balanced crystalloids.^[4]^ This implies that considering most ED patients do not require ICU admission, balanced crystalloids could offer significant advantages in promoting patient recovery from illness.^[4]^

Despite the benefits, clinicians are sometimes hesitant to administer balanced crystalloids in the ED due to potential hyperkalemia development, especially in patients with decreased kidney function.^[5,6]^ Studies in the operating room indicate that balanced crystalloids yield better outcomes in electrolyte control than saline. However, most of these studies were based on patients with end-stage renal disease during kidney transplantation surgery, and the outcomes were not consistent across studies.^[7–14]^ Additionally, no studies have evaluated changes in plasma potassium levels of ED patients with chronic kidney disease (CKD).

Thus, our study aimed to evaluate changes in potassium levels by administering balanced crystalloid Plasma Solution A (a generic of Plasma-Lyte) to ED patients with CKD.

## 2. Methods

### 2.1. Study design

This study was a retrospective cohort study aimed at comparing changes in potassium levels between patients with and without CKD, who received Plasma Solution A. Plasma Solution A contains 140 mmol/L of sodium, 5 mmol/L of potassium, 1.5 mmol/L of magnesium, 98 mmol/L of chloride, 27 mmol/L of acetate, and 23 mmol/L of gluconate, which is the equivalent of Plasma-Lyte. We conducted a review of adult patients aged 19 years and above who visited the Seoul National University Bundang Hospital ED between January 1, 2019, and June 30, 2021. The institutional review board at Seoul National University Bundang Hospital approved this study (IRB No. B-2008-630-109), and informed consent was waived.

### 2.2. Inclusion and exclusion criteria

In this study, patient eligibility was determined by several inclusion and exclusion criteria. Eligible patients were those who received intravenous Plasma Solution A and underwent an electrolyte follow-up test, specifically testing for potassium levels within 24 hours of administration. Patients with a known diagnosis of end-stage renal disease on dialysis were excluded from the study, as they rarely require massive fluid administration. Patients who were on a diet or received other potassium-containing fluids, such as parenteral nutrition, potassium chloride in 5% dextrose and sodium chloride injection, potassium chloride, or potassium phosphate, were also excluded. In addition, patients who received intravenous insulin or beta-blockers were excluded, as these treatments can influence potassium levels by shifting potassium between intracellular and extracellular spaces. Finally, patients who received polystyrene (kalimate) or intravenous furosemide were excluded, as polystyrene is a potassium binding resin typically used to reduce potassium levels and furosemide can induce renal loss of potassium. During the study period, all oral medications that have the potential to impact potassium levels, including angiotensin receptor blockers, angiotensin-converting enzyme inhibitors, thiazide, spironolactone, and furosemide, were withheld.

### 2.3. Data collection and subgroups

The vital signs data, blood urea nitrogen (BUN) levels, creatinine (Cr) levels, initial and follow-up electrolyte levels, as well as the medical histories of underlying conditions such as hypertension, diabetes mellitus, and CKD, were obtained from the electronic health records of our hospital. CKD diagnosis was confirmed by a nephrologist at the same hospital, and patients were classified according to the Kidney Disease: Improving Global Outcomes guideline. The classification system includes 5 grades based on estimated glomerular filtration rate (eGFR): grade 1 (eGFR ≥ 90 mL/minute/1.73 m^2^), grade 2 (eGFR 60–89 mL/minute/1.73 m^2^), grade 3 (eGFR 30–59 mL/minute/1.73 m^2^), grade 4 (eGFR 15–29 mL/minute/1.73 m^2^), and grade 5 (eGFR < 15 mL/minute/1.73 m^2^).^[15]^ The eGFR was calculated using the Modification of Diet in Renal Disease formula: (estimated GFR MDRD = 186 × serum creatinine [mg/dL] − 1.154 × age − 0.203 × [0.742 if female]).^[16]^

### 2.4. Outcome

The primary outcome of this study involved the evaluation of changes in potassium levels within 24 hours after administering Plasma Solution A to patients with CKD.

### 2.5. Statistical analysis

In this study, we presented the clinical characteristics of patients by means of means and standard deviations (SDs) for normally distributed variables, and counts (with percentage) for categorical variables. The enrolled patients were categorized into 2 groups based on their kidney function: the CKD group and the non-CKD (normal kidney function) group. To minimize potential confounding variables between these groups, we performed a 1:1 propensity score matching analysis, matching patients based on sex, age, height, weight, hypertension or diabetes mellitus, vital signs, sodium, potassium, chloride, administered volume of Plasma Solution A. We assessed initial and follow-up levels of electrolytes for each group. Potassium level changes in patients were evaluated using a *t* test and paired *t* test. We conducted statistical analysis using IBM SPSS ver.27.0 (IBM Co., Armonk, NY).

## 3. Results

### 3.1. Participant characteristics

**Figure 1.**
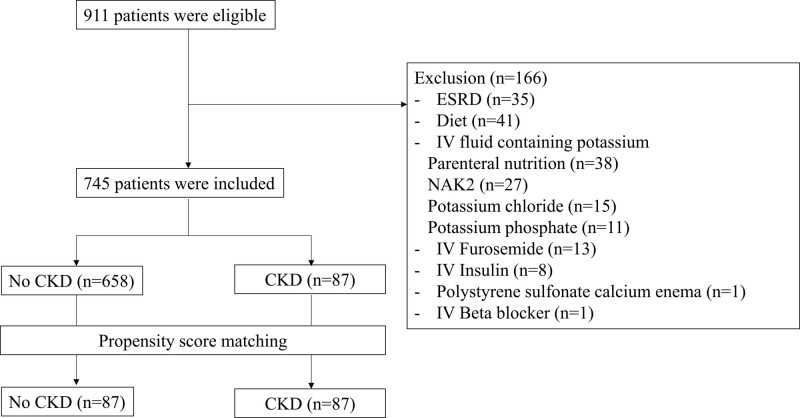
Patient enrollment. ESRD = end-stage renal disease, CKD = chronic kidney disease, IV = intravenous, PN = parenteral nutrition; NAK2, Dextrose 5% water with Na + 77 mEq and K + 20 mEq.

In this study, we reviewed a total of 911 patients, of which 745 patients were included. The baseline characteristics of the patients prior to propensity score matching are presented in Table 1. We observed significant differences in underlying hypertension, BUN, Cr, eGFR, and potassium levels. We performed 1:1 propensity score matching on 87 patients with CKD and 658 patients without CKD, based on sex, age, height, weight, hypertension or diabetes mellitus, vital signs, sodium, potassium, chloride, and administered volume of Plasma Solution A (Fig. 1). After propensity score matching, the CKD and non-CKD groups had a mean SD age of 64.5 (15.7) and 67.1 (15.2) years (*P* = .768), mean SD Cr levels of 2.3 (2.6) and 1.8 (1.7) mg/dL (*P* = .087), mean SD BUN levels of 37.2 (22.1) and 35.4 (25.3) mg/dL, mean SD potassium levels of 4.3 (0.6) and 4.2 (0.7) mmol/L (*P* = .396), and mean SD volume of Plasma Solution A administered of 28.7 (21.0) and 30.7 (22.2) mL/kg, respectively (Table 1).

**Table 1:** Patients background characteristics before and after propensity score matching.

Factor	Before matching			After matching		
	Without CKD (n = 658)	With CKD (n = 87)	*P* value	Without CKD (n = 87)	With CKD (n = 87)	*P* value
Man sex, n (%)	321 (48.8)	37 (38.5)	.077	40 (41.7)	37 (38.5)	.768
Age, mean SD, yr	63.1 (15.9)	64.5 (15.7)	.432	67.1 (15.2)	64.5 (15.7)	.234
Height, mean SD, cm	163.1 (9.4)	163.4 (8.2)	.800	163.5 (11.0)	163.4 (8.2)	.959
Weight, mean SD, kg	61.2 (13.3)	61.9 (12.9)	.653	60.9 (12.4)	61.9 (12.9)	.592
Hypertension, n (%)	283 (43.0)	51 (53.1)	.079	59 (61.5)	51 (53.1)	.307
Diabetes mellitus, n (%)	227 (34.5)	50 (52.1)	.001	47 (49.0)	50 (52.0)	.773
Systolic blood pressure, mean SD, mm Hg	130.0 (27.0)	125.8 (26.4)	.145	125.6 (35.4)	125.8 (26.4)	.967
Diastolic blood pressure, mean SD, mm Hg	71.7 (17.6)	68.8 (17.7)	.126	66.6 (20.8)	68.8 (17.7)	.431
Mean blood pressure, mean SD, mm Hg	92.8 (20.2)	90.9 (20.6)	.394	88.1 (25.2)	90.9 (20.6)	.401
Pulse rate, mean SD/min	94.6 (21.3)	92.1 (19.1)	.277	93.5 (21.2)	92.1 (19.1)	.620
Respiratory rate, mean SD/min	20.0 (5.0)	19.3 (5.3)	.263	19.8 (3.8)	19.3 (5.3)	.518
Body temperature, mean SD, °C	37.1 (1.1)	36.8 (1.1)	.009	36.8 (1.1)	36.8 (1.1)	1.000
BUN, mean SD, mg/dL	20.5 (14.1)	37.2 (22.1)	<.001	35.4 (25.3)	37.2 (22.1)	.613
Creatinine, mean SD, mg/dL	1.0 (0.8)	2.3 (2.6)	<.001	1.8 (1.7)	2.3 (2.6)	.087
eGFR-MDRD, mean SD, mL/min/1.73 m^2^	88.2 (40.5)	50.7 (39.2)	<.001	54.6 (41.4)	50.7 (39.2)	.496
Sodium, mean SD, mmol/L	138.0 (4.9)	138.3 (4.9)	.489	138.3 (5.2)	138.3 (4.9)	.952
Potassium, mean SD, mmol/L	4.1 (0.6)	4.3 (0.6)	.029	4.2 (0.7)	4.3 (0.6)	.396
Chloride, mean SD, mmol/L	102.7 (5.9)	103.8 (5.9)	.098	103.8 (6.3)	104.0 (6.2)	.679
PSA, mean SD, mL/kg	29.7 (21.3)	28.7 (21.0)	.672	30.7 (22.2)	28.7 (21.0)	.525
Time interval, mean SD, h	14.1 (4.7)	13.2 (4.5)	.338	14.8 (5.3)	13.2 (4.5)	.253

BUN = blood urea nitrogen, CKD = chronic kidney disease, eGFR = estimated glomerular filtration rate, PSA = plasma solution A, SD = standard deviation.

Among the cohort of patients with CKD, 11 patients exhibited an eGFR of at least 90 mL/minute/1.73 m^2^, while 16 patients had an eGFR of 60 to 90 mL/minute/1.73 m^2^. Furthermore, 39 patients had an eGFR of 30 to 60 mL/minute/1.73 m^2^, 14 patients had an eGFR of 15 to 30 mL/minute/1.73 m^2^, and 7 patients had an eGFR <30 mL/minute/1.73 m^2^. The mean SD eGFR levels for each group were 132.2 (49.1), 70.7 (6.5), 42.9 (9.7), 25.3 (13.1), and 7.4 (4.0) mL/minute/1.73 m^2^, respectively. Additionally, the mean SD potassium levels were 3.8 (0.7), 4.2 (0.5), 4.4 (0.6), 4.4 (0.3), and 4.5 (0.6) mmol/L, respectively. The mean SD volume of Plasma Solution A administered per body weight (mL/kg) for each group were 24.8 (9.0), 21.8 (12.6), 33.7 (27.3), 31.2 (17.2), and 24.6 (19.7), respectively.

### 3.2. Changes in potassium levels

We assessed the changes in the electrolyte levels for each group. The mean SD potassium levels decreased by 0.2 (0.5) mmol/L in the non-CKD group (*P* < .001) and 0.1 (0.5) mmol/L in the CKD group (*P* < .001) (Table 2). However, no significant differences were observed in the electrolyte levels changes between the CKD and non-CKD groups at the follow-up test within 24 hours of Plasma Solution A administration (Table 3).

**Table 2:** Changes in electrolyte at follow up test within 24 h.

		Initial level, mean SD	Follow-up level, mean SD	Difference, mean SD	95% CI	*P* value
Without CKD (n = 87)	Sodium (mmol/L)	138.3 (5.2)	140.0 (4.9)	1.7 (2.8)	0.94–2.48	<.001
	Potassium (mmol/L)	4.2 (0.7)	4.0 (0.6)	−0.2 (0.5)	−0.39 to −0.11	<.001
	Chloride (mmol/L)	103.8 (6.3)	105.3 (5.7)	1.9 (3.2)	0.50–1.89	<.001
With CKD (n = 87)	Sodium (mmol/L)	138.3 (4.9)	140.2 (4.0)	1.9 (3.2)	1.18–2.53	<.001
	Potassium (mmol/L)	4.3 (0.6)	4.1 (0.6)	−0.1 (0.5)	−0.22 to −0.30	.029
	Chloride (mmol/L)	104.0 (6.2)	105.9 (5.4)	2.0 (3.1)	1.41–2.72	<.001

BUN = blood urea nitrogen, CI = confidence interval, CKD = chronic kidney disease, SD = standard deviation.

**Table 3:** The differences in electrolyte levels changes between patients with or without CKD.

	Without CKD (n = 87)	With CKD (n = 87)	95% CI	*P* value
	Difference, mean SD	Difference, mean SD		
Sodium, mean SD, mmol/L	1.7 (2.8)	1.9 (3.2)	−1.16–0.87	.728
Potassium, mean SD, mmol/L	−0.2 (0.5)	−0.1 (0.5)	−0.25–0.05	.452
Chloride, mean SD, mmol/L	1.9 (3.2)	2.0 (3.1)	−1.82–0.07	.722

BUN = blood urea nitrogen, CI = confidence interval, CKD = chronic kidney disease, eGFR = estimated glomerular filtration rate, PSA = plasma solution A, SD = standard deviation.

Among patients with eGFR >90, the mean SD potassium levels decreased by 0.1 (0.1) mmol/L. In patients with eGFR between 60 and 90, the mean SD potassium level decreased by 0.1 (0.4) mmol/L. In patients with eGFR between 30 and 60, the mean SD potassium level decreased by 0.1 (0.5) mmol/L. Patients with eGFR between 15 and 30 exhibited a more significant decrease in mean SD potassium levels, with a decrease of 0.5 (0.4) mmol/L. Lastly, patients with eGFR <15 demonstrated a mean SD decrease in potassium levels of 0.2 (0.5) mmol/L. The potassium levels increased in 24 patients after the administration of Plasma Solution A, from 4.1 ± 0.6 mmol/L to 4.5 ± 0.5 mmol/L, but hyperkalemia (>5.5 mmol/L) did not occur in these patients despite Plasma Solution A administration.

## 4. Discussion

The Surviving Sepsis Campaign issued a recommendation to use balanced crystalloid instead of saline.^[17]^ A previous study conducted in an ED demonstrated that the administration of balanced crystalloid resulted in superior renal outcomes in comparison to saline.^[4]^ Despite these findings, some clinicians remain hesitant to use balanced crystalloids due to the potential risk of unforeseen hyperkalemia in patients with CKD.^[5,6]^ There have been studies conducted in operating room, including the study by Catherine et al, suggesting that balanced crystalloid is superior to normal saline in the regulation of electrolytes, including potassium, before and after surgery,^[11,14]^ but the literature regarding changes in potassium levels resulting from the administration of balanced crystalloids in ED patients is limited. In a previous study, the authors found that there were no hyperkalemia when balanced crystalloid was administered to patients with acute tubular dysfunction presenting to the ED,^[18]^ but this was in patients without CKD and no studies have been conducted in patients with CKD presenting to the ED. The findings of this study indicate that the administration of Plasma Solution A to patients with CKD does not appear to result in a significant increase in potassium levels. On the contrary, the administration of Plasma Solution A led to a decrease in potassium levels and an improvement in renal function in the studied population (see Table S1, Supplemental Digital Content, http://links.lww.com/MD/J717 which contains data on renal function and electrolyte changes in CKD patients, specifically including follow-up values for renal function). As such, Plasma Solution A, as a balanced crystalloid, may be a safe first-line fluid choice in the ED for patients with CKD without the risk of hyperkalemia.

There are several reasons to suggest that the change in potassium levels following the administration of Plasma Solution A is not significantly meaningful. Firstly, the quantity of potassium in 1 L of Plasma Solution A is only 4 mmol/L, which is a negligible amount when distributed throughout the body. Secondly, approximately 98% of the total body potassium is stored in cells, indicating that the intracellular to extracellular potassium shift has a much more substantial effect on serum potassium levels than the 4 mmol/L potassium content of Plasma Solution A. For example, saline administration may induce hyperchloremic acidosis, which prompts potassium to shift out of cells, increasing serum potassium levels. Thirdly, a potassium intake of 90 mmol/day is adequate for a healthy adult,^[19]^ which corresponds to roughly 4 mmol of potassium per hour. Therefore, 4 mmol/L of potassium in Plasma Solution A may be required to maintain healthy potassium levels.

This study had several limitations. Firstly, the follow-up tests for potassium electrolyte levels were conducted at various time intervals within 24 hours of administering Plasma Solution A, making it impossible to evaluate the hourly effect of balanced crystalloids in ED patients. Secondly, due to the limited availability of patient records on the stage of CKD, the effect of Plasma Solution A on the degree of CKD could not be determined. Thirdly, our findings indicate that levels of Cr decreased and eGFR increased in all patients with reduced renal function following the administration of Plasma Solution A. The infusion of Plasma Solution A improved perfusion, resulting in a further reduction in the risk of hyperkalemia. However, since we did not assess the patients’ ultimate diagnoses of kidney diseases, it remains unclear whether the use of Plasma Solution A can be safely administered to patients who present to the ED with conditions unrelated to dehydration-related diseases. Finally, this was a retrospective study with a small sample size, and a prospective study with a larger sample size is necessary in the future to validate the findings of this study.

## 5. Conclusion

The results of this study suggest that the administration of approximately 2 L of balanced crystalloid intravenously may not significantly increase potassium levels within 24 hours in patients with CKD. Therefore, it is possible that balanced crystalloids could be considered as a safe standard fluid in EDs for patients with CKD.

## Author contributions

**Conceptualization:** Sungsig Kong, Hyuksool Kwon.

**Formal analysis:** Sungsig Kong.

**Investigation:** Sungsig Kong.

**Methodology:** Sungsig Kong.

**Supervision:** Hyuksool Kwon.

**Writing – original draft:** Sungsig Kong.Writing – review & editing: Hyuksool Kwon.

## Supplementary Material


