# Supplementary material for: Potassium level changes in chronic kidney disease patients following balanced crystalloid administration in the emergency department
Source: Medicine (Baltimore). 2023 Sep 29;102(39):e35026. doi: 10.1097/MD.0000000000035026 (PMC10545324; doi:10.1097/MD.0000000000035026)
Supplement: Supplementary file 1 [file medi-102-e35026-s001.docx]

Supplement table 1. Renal function and electrolyte changes in patients with renal function follow-up values assessed within 24 hours, after undergoing 1:1 propensity score matching

|  |  | | **Initial level, mean (SD)** | **Follow-up level, mean (SD)** | **Difference,**  **mean (SD)** | **95% CI** | **P-value** |
| --- | --- | --- | --- | --- | --- | --- | --- |
| Without CKD  (n=25) | Sodium (mmol/L) | | 137.6 (4.5) | 138.9 (2.6) | 1.3 (2.6) | 0.22 to -2.34 | 0.020 |
|  | Potassium (mmol/L) | | 4.5 (0.4) | 4.1 (0.4) | 0.4 (0.4) | -0.53 to -0.23 | <0.001 |
|  | Chloride (mmol/L) | | 104.3 (5.6) | 105.1 (2.3) | 0.8 (2.3) | -0.20 to 1.72 | 0.114 |
|  | BUN (mg/dl) | | 18.5 (9.9) | 17.5 (4.3) | 1.0 (4.2) | -2.80 to 0.72 | 0.235 |
|  | Creatinine (mg/dl) | | 0.9 (0.4) | 0.8 (0.2) | 0.7 (0.2) | -0.16 to -0.02 | 0.109 |
|  | eGFR (ml/min) | | 95.2 (38.1) | 102.0 (28.3) | 6.8 (28.3) | -4.86 to 18.5 | 0.240 |
| With CKD  (n=25) | Sodium (mmol/L) | | 137.6 (4.4) | 140.2 (2.6) | 2.6 (2.5) | 1.55 to 3.65 | <0.001 |
|  | Potassium (mmol/L) | | 4.5 (0.6) | 4.2 (0.3) | 0.3 (0.4) | -0.49 to -0.14 | 0.001 |
|  | Chloride (mmol/L) | | 104.7 (7.2) | 106.9 (3.4) | 2.2 (3.4) | 0.81 to 3.60 | 0.003 |
|  | BUN (mg/dl) | | 33.7 (17.0) | 32.0 (6.9) | 1.7 (6.9) | -4.52 to 1.16 | 0.234 |
|  | Creatinine (mg/dl) | | 2.1 (2.4) | 1.9 (0.4) | 0.2 (0.4) | -0.39 to -0.05 | 0.014 |
|  | eGFR (ml/min) | | 47.4 (24.6) | 57.4 (17.1) | 10.0 (17.1) | 2.95 to 17.09 | 0.007 |
|  | | BUN, blood urea nitrogen; CI, confidence interval; CKD, chronic kidney disease; eGFR, estimated glomerular filtration rate; SD, standard deviation | | | | | |
